# Association between procalcitonin and mortality in patients with necrotizing fasciitis: A single-center retrospective study

**DOI:** 10.1016/j.jpra.2026.03.014

**Published:** 2026-03-07

**Authors:** Alperen S. Bingoel, Khaled Dastagir, Nele Schmitt, Najib Dastagir, Mustafa Salim, Peter M. Vogt, Doha Obed

**Affiliations:** a Division of Plastic Surgery, Department of Trauma, Orthopedic and Plastic Surgery, University Medical Center Göttingen, Robert-Koch-Str. 40, 37075 Göttingen, Germany; b Department of Plastic, Aesthetic, Hand and Reconstructive Surgery, Burn Center, Hannover Medical School, Carl-Neuberg-Str. 1, 30625 Hannover, Germany; c Department of Human Genetics, Hannover Medical School, Hannover, Germany

**Keywords:** Necrotizing fasciitis, Procalcitonin, Mortality, Reconstructive surgery, Biomarker

## Abstract

**Background:**

Necrotizing fasciitis (NF) is a rapidly progressing, life-threatening soft tissue infection. Procalcitonin (PCT) has emerged as a promising biomarker for early diagnosis and prognosis in severe infections.

**Methods:**

We performed a retrospective analysis of 62 patients with confirmed NF treated at a high-volume Department of Plastic Surgery between January 2005 and May 2024. Clinical parameters, laboratory values, and surgical outcomes were evaluated. Patients were stratified into survivors and non-survivors.

**Results:**

Elevated PCT levels at admission were significantly associated with in-hospital mortality (*p* = 0.0329), whereas other parameters showed no statistical significance. Skin grafting was the most common reconstructive procedure. No significant association was found between the type of reconstructive surgery and survival.

**Conclusion:**

Serum PCT levels at admission may serve as a valuable prognostic marker in NF. Once infection control is achieved, reconstructive surgical interventions appear to be safe and do not influence survival.

## Introduction

Necrotizing fasciitis (NF) is an uncommon disease that is characterized by progressive infection of the subcutaneous and muscle tissue as well as the fascial system with necrosis. Rapid diagnosis and treatment are crucial for survival in this potentially life-threatening condition. Limb loss, systemic complications such as sepsis, and multiple organ failure with fatal outcome are observed frequently.^1^^,^^2^

Despite advances in critical care, surgery, and antimicrobial therapy, mortality rates associated with this condition range from 6 % to 33 %.^3^ Notably, some studies have reported alarmingly high rates of up to 70 % depending on causative pathogens, pathophysiology and the timing of treatment initiation.^4^ Therefore, timely diagnosis as well as correct timing of plastic surgical reconstruction remains a major challenge.

The clinical manifestations are often non-specific and inconspicuous, further complicating early diagnosis. The Laboratory Risk Indicator for Necrotizing Fasciitis (LRINEC) score has become a pivotal diagnostic tool in identifying cases of NF, incorporating various laboratory parameters.^5^ While the LRINEC score provides a straightforward, non-invasive approach to early diagnosis, important publications suggest that a distinction should be made between diabetes and non-diabetic populations as well as that it may not be an accurate tool for distinguishing between NF and cellulitis.^6^^,^^7^ In addition, some researchers advocate for refinement of the LRINEC score to improve the diagnostic process.^8^

C-reactive protein (CRP) and leukocyte elevation are present but non-specific in severe infections, while procalcitonin (PCT) is more specific to bacterial infection and sepsis.^9^ A meta-analysis by Arora et al. demonstrated that PCT levels measured in the early stages of sepsis are significantly lower among survivors compared to non-survivors.^10^

Furthermore, several studies have demonstrated that PCT can exhibit high sensitivity and specificity in the detection of fulminant NF.^11^, ^12^, ^13^

However, the reliable diagnosis and management of NF are further complicated due to several factors, including the absence of widely accepted biomarkers, frequently ambiguous or even absent clinical symptoms, limited experience among medical personnel, and the lack of standardized diagnostic criteria. In addition to rapid debridement and infection control, reconstructive surgery plays a pivotal role in restoring tissue integrity and function. Determining the optimal timing and strategy for plastic-reconstructive procedures remains a complex aspect of NF management.

The objective of this study was to evaluate a cohort of patients treated for NF at a university hospital with a high volume of plastic surgery cases. Specifically, the study aimed to identify patterns in clinical presentation, laboratory values, and surgical management. Particular emphasis was placed on assessing the prognostic value of PCT and the outcomes of reconstructive interventions.

## Materials and methods

### Patient selection and data collection

We conducted a retrospective review of patients treated for necrotizing fasciitis between January 2005 and May 2024 in the Department of Plastic Surgery at a university hospital. Eligible patients were identified using the hospital information systems (SAP, Walldorf, Germany; m.life, medisite, Hannover, Germany), by searching for the International Classification of Diseases (ICD) codes M72.6 and M49.80. The following parameters were collected: age, sex, comorbidities, smoking history, body mass index (BMI), anatomical site of infection, secondary transfer from another hospital, blood culture results, presence of sepsis, Laboratory Risk Indicator for Necrotizing Fasciitis (LRINEC) score at admission, leukocyte count at admission and during the clinical course, C-reactive protein (CRP) at admission and during the clinical course, and procalcitonin (PCT) at admission and during the clinical course.

Surgical data included the number of operative procedures, number of negative pressure wound therapy (NPWT) cycles, creation of an anus praeter, duration of intensive care unit (ICU) stay, total hospital stay, mortality, and types of reconstructive procedures performed. In all cases, standard protocols were followed, including preservation of pathological samples for further diagnostic evaluation.

### Statistics

Patients were divided into two groups for comparison: survivors and non-survivors. Continuous variables are presented as mean values with standard deviations, and categorical variables are reported as frequencies and percentages. For group comparisons, the Student’s *t*-test was used for continuous variables and the binomial test for categorical variables. A *p*-value of < 0.05 was considered statistically significant.

All statistical analyses were performed using GraphPad Prism (Version 9.3.1 (350) for macOS Monterey, GraphPad Software, San Diego, CA, USA).

## Results

In total, 62 patients could be identified in the analyzed period. The mean age of patients at admission was 56.68 ± 12.30 years. Gender distribution was recorded, with 42 patients identified as male, corresponding to 67.74 % of the total population. Patients were further stratified into age groups: 11 patients (17.74 %) were between 25 and 44 years old, 37 patients (59.68 %) between 45 and 64 years, and 14 patients (22.58 %) were 65 years of age or older.

The presence of active smoking was documented, with 18 patients (29.03 %) identified as active smokers. Body mass index (BMI) was recorded for each patient, with a calculated mean of 30.20 ± 10.70 kg/m².

Comorbid conditions were systematically documented. Among the study population, 32 patients (51.61 %) had arterial hypertension, 27 (43.55 %) had diabetes mellitus, and 5 (8.06 %) were diagnosed with peripheral arterial disease. Coronary artery disease was present in 7 patients (11.29 %), while chronic obstructive pulmonary disease (COPD) was found in 7 patients (11.29 %). Additionally, 10 patients (16.13 %) suffered from cardiac arrhythmia, 8 (12.90 %) had heart failure, and 13 (20.97 %) were diagnosed with renal insufficiency. The anatomical location of the affected area was classified into seven categories. In the cohort under study, no patient exhibited NF in the head and neck region.

A total of 8 patients (12.90 %) presented with lesions on the trunk, 9 patients (14.52 %) in the abdominal area, 27 patients (43.55 %) in the perineal region, 8 patients (12.90 %) on the upper extremity, 12 patients (19.35 %) on the lower leg, and 19 patients (30.65 %) on the upper leg.

Additional clinical data were also collected. A total of 24 patients (38.71 %) were transferred to the facility from other medical centers secondarily. Blood cultures were obtained on admission, and 17 patients (27.42 %) showed positive results. Sepsis was diagnosed in 30 patients, accounting for 48.39 % of the cohort.

The LRINEC score at admission was calculated for all patients and yielded a mean value of 5.71 ± 2.95. The leukocyte count at admission was 14.38 ± 10.34 × 10⁹/L. The progression of leukocyte levels during the clinical course was monitored, with a mean of 21.98 ± 17.16 × 10⁹/L recorded.

C-reactive protein (CRP) levels at admission averaged 194.47 ± 136.48 mg/dL, with a mean follow-up value of 177.94 ± 138.50 mg/dL over the course of treatment. PCT levels were also measured, showing a mean of 13.68 ± 34.77 µg/L at admission and of 5.63 ± 20.09 µg/L during follow-up.

The mean length of stay in the intensive care unit (ICU) was 30.21 ± 39.46 days, while the mean total hospital stay was 59.73 ± 44.64 days. The mortality rate within the cohort was found to be 16.13 % (10 cases). A comprehensive list of all the medical parameters collected is provided in Table 1.

**Table 1:** Clinical characteristics.

Variable	Total (*n* = 62)	Survivors (*n* = 52)	Non-Survivors (*n* = 10)	*p*-value
Age (years) (mean ± SD)	56.68 ± 12.30	56.19 ± 12.28	59.1 ± 13.47	0.5016
Male Gender, *n* (%)	42 (67.74)	34 (65.38)	8 (80)	>0.9999
Age Group, *n* (%)				
25–44 45–64 ≥65	11 (17.74)	9 (17.31)	2 (20)	>0.9999
	37 (59.68)	32 (61.54)	5 (50)	0.4134
	14 (22.58)	11 (21.15)	3 (30)	0.7499
Comorbidities, *n* (%)				
Hypertension Diabetes Peripheral arterial disease Coronary artery disease COPD Arrhythmia Heart insufficiency Renal insufficiency	32 (51.61)	26 (50)	5 (50)	0.8220
	27 (43.55)	22 (42.31)	4 (40)	0.8059
	5 (8.06)	5 (9.62)	0 (0)	0.5904
	7 (11.29)	5 (9.62)	2 (20)	0.6330
	7 (11.29)	5 (9.62)	2 (20)	0.6330
	10 (16.13)	9 (17.31)	1 (10)	0.6980
	8 (12.90)	6 (11.54)	2 (20)	0.6645
	13 (20.97)	11 (21.16)	2 (20)	>0.9999
Active smokers, *n* (%)	18 (29.03)	15 (28.85)	3 (30)	>0.9999
BMI (kg/m²) (mean ± SD)	30.20 ± 10.70	30.23 ± 11.5	30.91 ± 14	0.8692
Area affected, *n* (%)				
Head and neck Trunk Abdomen Perineum Upper extremity Lower leg Upper Leg	0 (0)	0 (0)	0 (0)	-
	8 (12.90)	6 (11.54)	2 (20)	0.6645
	9 (14.52)	7 (13.46)	2 (20)	>0.9999
	27 (43.55)	24 (46.15)	3 (30)	0.3378
	8 (12.90)	6 (11.54)	2 (20)	0.6645
	12 (19.35)	10 (19.23)	1 (10)	0.7047
	19 (30.65)	15 (28.85)	4 (40)	>0.9999
Transfer from another center, *n* (%)	24 (38.71)	19 (36.54)	5 (50)	>0.9999
Positive blood cultures *n* (%)	17 (27.42)	14 (26.92)	3 (30)	>0.9999
Sepsis *n* (%)	30 (48.39)	23 (44.23)	7 (70)	0.6483
LRINEC-score on admission (mean ± SD)	5.71 ± 2.95	5.60 ± 3.06	6.3 ± 2.19	0.9148
WBC count (x 10^9^/L on admission (mean ± SD)	14.38 ± 10.34	13.99 ± 10.51	16.37 ± 9.15	0.5067
WBC count (x 10^9^/L) clinical course (mean ± SD)	21.98 ± 17.16	22.25 ± 18.40	20.64 ± 8.21	0.7880
CRP (mg/dL) on admission (mean ± SD)	194.47 ± 136.48	186.86 ± 134.71	233.33 ± 138.87	0.3240
CRP (mg/dL) clinical course (mean ± SD)	177.94 ± 138.50	167.42 ± 143.44	231.61 ± 96.32	0.1812
PCT (µg/L) on admission (mean ± SD)	13.68 ± 34.77	9.19 ± 23.52	33.12 ± 59.86	**0.0329**
PCT (µg/L) clinical course (mean ± SD)	5.63 ± 20.09	5.63 ± 22.13	4.9 ± 4.39	0.9181

Bold values indicate statistical significance (p < 0.05).

COPD: Chronic obstructive pulmonary disease; CRP: C-reactive protein; PCT: Procalcitonin; SD: Standard deviation; WBC: White blood cell.

Regarding surgical management, patients underwent an average of 8.16 ± 9.17 operative procedures, including 3.32 ± 5.24 cycles of negative pressure wound therapy (NPWT). Additionally, 22 patients (35.48 %) required reconstructive procedures involving the creation of an anus praeter. The most frequently performed procedure was skin grafting (*n* = 39; 62.90 %), followed by flap reconstruction (*n* = 23; 37.10 %) and secondary wound closure (*n* = 20; 32.26 %). Details of the reconstructive interventions are summarized in Table 2.

**Table 2:** Surgical treatment and outcome.

Variable	Total (*n* = 62)	Survivors (*n* = 52)	Non-survivors (*n* = 10)	*p*-value
Treatment modality, *n* (%)				
Split thickness skin graft Full thickness skin graft Free flap Amputation Secondary closure	39 (62.90)	35 (67.31)	4 (40)	0.1608
	1 (1.61)	1 (1.92)	0 (0)	>0.9999
	23 (37.10)	21 (40.38)	2 (20)	0.2930
	4 (6.45)	4 (7.69)	0 (0)	>0.9999
	20 (31.75)	17 (32.69)	3 (30)	0.7818
Surgical intervention rate, mean ± SD	8.16 ± 9.17	8.25 ± 9.29	8.22 ± 8.50	0.9925
NWPT cycles, mean ± SD	3.32 ± 5.24	3.65 ± 5.50	1.6 ± 3.04	0.2586
Anus praeter rate, *n* (%)	22 (35.48)	19 (36.54)	3 (30)	0.5994
Length of ICU stay (days), mean ± SD	30.21 ± 39.48	33.44 ± 41.70	13.4 ± 16.87	0.1419
Length of hospital stay (days), mean ± SD	59.73 ± 44.64	62.02 ± 45.22	47.8 ± 39.39	0.3573
Mortality, *n* (%)	10 (16.13)	0 (0)	10 (100)	-

ICU: Intensive care unit; NPWT: Negative pressure wound therapy; SD: Standard deviation.

When comparing survivors and non-survivors, a statistically significant difference was observed in PCT levels on admission (*p* = 0.0329), while all other parameters showed no significance between the groups.

**Figure 1:**
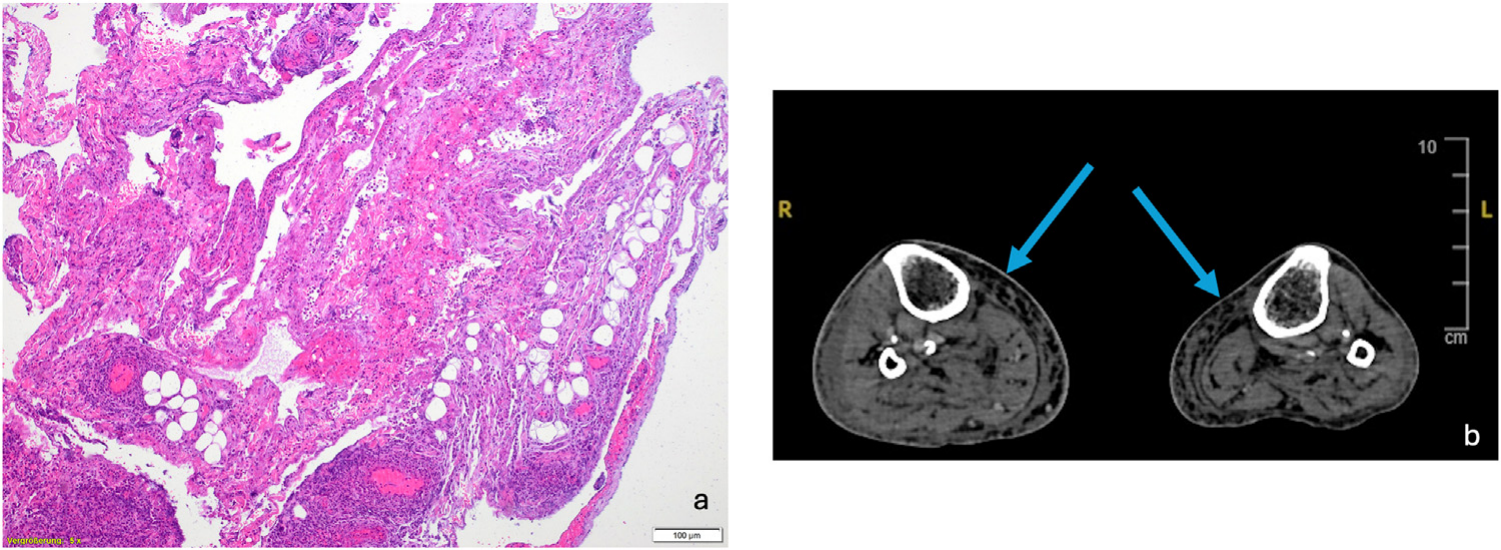
(a) Hematoxylin and eosin (H&E)-stained histological section of infected soft tissue (5 × magnification), demonstrating extensive, diffusely distributed mixed inflammatory infiltrates, necrotic debris, and disrupted architecture indicative of severe infection. (b) Axial CT image of the lower extremities showing subcutaneous gas accumulation (blue arrows), a radiological hallmark suggestive of necrotizing soft tissue infection (NSTI).

Figure 1 presents the preoperative findings in CT scan with gas inclusions and results of the corresponding histopathological evaluations.

## Discussion

This retrospective study evaluated clinical, laboratory, and surgical data from 62 patients diagnosed with necrotizing fasciitis (NF) at a tertiary plastic surgery center. Notably, procalcitonin (PCT) levels at admission were the only parameter that showed a statistically significant difference between survivors and non-survivors (*p* = 0.0329). This suggests a potential role for PCT as an early prognostic marker. Despite the significant advancements in diagnostics, surgical management, and intensive care therapies that have transpired in recent decades, the mortality rate associated with NF remains high. This is largely attributable to a delay in diagnosis and timely surgical therapy.^3^

The diverse pathogens implicated in NF, together with the resultant pathophysiology and associated host factors, are critical determinants of the disease process.^4^^,^^14^ Key contributing factors include diabetes mellitus, smoking, and obesity.^15^

A multicenter study with 62 patients by Misiakos et al. showed an overall low mortality of 17.7 %.^1^ This is comparably low compared to the mortality rate found in this study (16.13 %). The observed outcomes were attributable to the efficiency of the diagnostic process and the implementation of assertive surgical interventions. The mean interval from patient admission to surgical intervention was reported to be 12.8 h. It was observed that cases that could only be operated on 24 h later exhibited a poorer clinical outcome. They also stated that in their patient cohort, the majority exhibited moderate scores of 6–8, with only 25.8 % demonstrating scores above ≥9. It is worth noting that the laboratory chemical analysis of PCT was not carried out in the study.

The LRINEC score offers a non-invasive tool; nevertheless, it may also demonstrate a diagnostic gap. Neeki et al. investigated the use of the LRINEC score in patients to differentiate between cellulitis and NF.^6^ Among the findings was the LRINEC score for NF demonstrating a high rate of false negatives. They suggested that the LRINEC score may not serve as a reliable tool for risk stratification in NF. PCT was not referenced in this work, either.

The clinical value of a biomarker-based approach lies in its potential to rapidly identify and prioritize high-risk cases for prompt diagnostic imaging and, when necessary, surgical intervention. The findings of this study indicate that elevated PCT levels at the time of admission are associated with an increased risk of in-hospital mortality, supporting its potential role as a prognostic marker. Misdiagnosis leads to high mortality, which is why rapid identification of NF is crucial. Although PCT is not specific for the presence of NF, it can be pivotal in initiating life-saving surgical procedures, potentially even in cases of false-positive results. Numerous studies have demonstrated that serum PCT is a well-established and clinically relevant biomarker for the diagnosis and management of critically ill patients.^16^, ^17^, ^18^

Specifically in the context of NF, several studies have highlighted a growing consensus on the utility of serum PCT as a biomarker, with potential to aid in early diagnosis and serve as a prognostic indicator. A comprehensive review by Zil-E-Ali et al. suggested that PCT may serve as a valuable biomarker for the early detection of disease, assessment of severity, and prediction of clinical outcomes. The authors recommended measuring serum PCT levels in all patients presenting to the emergency department with suspected soft tissue infection or NF.^12^

In a prospective study involving 38 patients with necrotizing soft tissue infections, Friederichs et al. identified the PCT ratio between postoperative day 1 and day 2 as a reliable indicator of effective surgical control of the infectious source.^11^ A ratio exceeding 1.14 was significantly correlated with clinical improvement, demonstrating a sensitivity of 83.3 % and a specificity of 71.4 %.

In our clinical approach, PCT trends and absolute levels are actively incorporated into clinical assessment and therapeutic decision-making, consistent with core clinical principles. Significant elevations in serum PCT are considered as an additional criterion for determining the need for further surgical intervention. In contrast to the study by Friederichs et al., our investigation did not specifically examine the kinetics of PCT levels in the preoperative or postoperative period.

A notable retrospective study by Al-Thani et al. analyzed admission PCT levels in a cohort of 62 patients, stratifying them into four risk categories.^19^ Their findings demonstrated a clear association between initial PCT levels and both the likelihood of developing septic shock and the severity of NF. A PCT threshold of 5.6 ng/mL was identified as predictive of septic shock, with a sensitivity of 81 % and a specificity of 67 %. Furthermore, a strong correlation was observed between PCT levels and the SOFA score.

A case-control study by Kishino et al. examined serum PCT as a biomarker to distinguish necrotizing fasciitis from cellulitis.^20^ The results support PCT as a valuable marker with an area under the ROC curve of 0.928 and an optimal cutoff of 1.0 ng/mL (88 % sensitivity, 89 % specificity). Interestingly, PCT outperformed the LRINEC Score (AUC = 0.846).

Our findings corroborate the conclusions of Al-Thani and Kishino, which demonstrate that the utilization of PCT levels at admission offers insights into the severity of the disease and facilitates the estimation of a fatal outcome.

Both Kishino and Kato et al. investigated the utility of PCT for differentiating NF from cellulitis.^21^ Kato et al. reported 100 % sensitivity and specificity for PCT in a short communication, although the study’s small sample size limits the generalizability of this result. These findings were corroborated by Vaibhavi et al., who conducted a prospective analysis of 30 patients and similarly demonstrated significantly elevated PCT levels in cases of NF compared to cellulitis (mean 4.89 µg/L vs. 0.34 µg/L, *p* < 0.001).^13^

Nevertheless, currently, no single laboratory parameter or panel of tests possesses sufficient diagnostic power to replace surgical inspection for identifying these infections.^4^ With respect to surgical management, which was also evaluated in the present study, no significant difference in survival was observed between the two groups in relation to the reconstructive procedure employed.

Early and repeated surgical debridement is critical in managing NF. Delays beyond 12–24 h significantly increase mortality.^1^^,^^14^^,^^22^ Aggressive intervention and tailored use of negative pressure wound therapy and reconstruction techniques improve outcomes. Reconstruction is required for soft tissue defects following debridement of non-vital tissue. In the present cohort, wound healing was achieved in the majority of cases, with surgical amputation necessary in 4 patients (6.45 %). A total of 21 free flap procedures were performed.

Importantly, there was no statistically significant association between the type of reconstructive procedure and survival, suggesting that once infection control is achieved, reconstructive choices can be tailored to the patient without influencing prognosis. Consistent with previous reports, this technique proved to be a safe and effective approach for managing such defects.^23^, ^24^, ^25^

A major strength of this study is the comprehensive analysis of both medical and surgical variables in a real-world clinical setting. However, limitations must be acknowledged. The study design cannot control confounders, and the small sample size may have limited statistical power, especially in subgroup analyses.

## Conclusion

Necrotizing fasciitis (NF) is a life-threatening condition in which patient survival depends critically on early diagnosis and prompt surgical intervention. The cohorts are often very heterogeneous, with varying pathogen profiles. We utilize PCT trends and absolute levels for clinical assessment and therapeutic decision-making. Emerging evidence supports the use of serum PCT levels as a valuable diagnostic tool. The findings of the present study further suggest that elevated serum PCT should be considered a supportive diagnostic marker in clinically suspected cases, as it may be associated with increased mortality risk. Regarding surgical management, plastic-reconstructive procedures such as skin grafting and flap reconstruction were found to be safe once infection control was achieved and did not impact overall survival in our cohort.

## Ethical approval

This study was conducted in accordance with the Declaration of Helsinki and approved by the institutional ethics committee of Hannover Medical School (approval number: 11152_BO_K_2023).

## Funding

There was no financial support of any kind contributing to this publication.

## Declaration of competing interest

None of the authors has any personal or institutional financial interests in any drugs, materials, or devices mentioned in this manuscript.
